# Carcinoma of the Spleen

**Published:** 1868-11-15

**Authors:** V. R. Bridges

**Affiliations:** Mattoon, Ill.


### CARCINOMA OF THE SPLEEN.

BY V. R. BRIDGES, M.D., MATTOON, ILL.

Harvey Tremble, native of Ohio, house carpenter, aged fifty-five years, ordinarily enjoyed good health, till during the winter of 1867–68, when he began to suffer somewhat from a sense of weariness, and aching in the left hypochondrium and across the stomach. While working at the bench would often have occasion to stop and straighten himself up, in order to relieve a "stitch or cramp," as he said, "in the short ribs;" otherwise his general health was unimpaired. On the 27th of April last, he called on Dr. W. E. Morris, of this place, for advice in relation to a vague uneasiness about his system; referred mainly to his left side and stomach. Dr. M. examined the case, prescribed an anodyne at bed-time, and gave him, perhaps, an antacid, with some general directions as to the care of his person, and he went away satisfied, but returned again in a few days with the same symptoms. The doctor prescribed again with similar results. He was finally called to his house, found him in bed somewhat debilitated, with variable appetite, some restlessness, and having pain in the left iliac fossa, and along the course of the descending colon, with constipated bowels. He prescribed an alterative, with a mustard plaster over the lower bowels. He was visited from day to day without observing but little change. He would occasionally have some fever, with manifest periodicity, when *Quinine* would be given with fair results. In the progress of the case he was seized with severe pain in the lumbar region, when anodynes and diuretics were given, and a blister applied over the kidneys. This afforded temporary relief. Finally, upon a more careful examination of the case, a remarkable prominence was observed in the left hypochondrium beneath the ribs, which were made to form the arc of a much greater circle than those of the opposite side. The tumor was of great density, and not very painful on pressure; no discoloration. He could rest quite easy in any position, his appetite somewhat improved, and he had no fever, except at intervals of from three to five days. The pain in the back ceased to trouble him much; some tenderness along the descending colon; bowels now easily controlled; no sickness at the stomach, but looks pale, or of a leaden color, and is somewhat emaciated.

Treatment.— Occasional alteratives and chalybeate tonics; counter irritation over tumor in the side; nutritious diet, and gentle exercise in the open air. At this time, May 27th, Dr. Morris expressed doubts as to the exact character of the disease, and desired me to visit the case with him. We each gave the case a thorough examination, and after watching it carefully and maturely considering the pathological changes, and interchanging opinions for a series of days, we came to the conclusion that the disease was malignant; in other words, was carcinoma of some of the viscera of the left side, but were not very decided as to the exact organ; were inclined to believe it was in the splenic arch of the colon. Having become satisfied of the malignant nature of the lesion, we could do no more than to give a palliative treatment. Not willing wholly to abandon a hope of benefiting the patient, we called together a number of the more prominent members of the profession in the city, for counsel. The consultation substantially sustained us in our opinion, though some were inclined to a different view, and that not without reasons which claimed our consideration. Of the result of the interview we advised the patient, and expressed to him freely, for the first time, our opinion, and advised him of the probable result of his case, and the course of treatment for the future. We expressed to him a willingness for him to call in any other counsel he might desire, or to make an entirechange if he wished, assuring him that we would watch the progress of the case, and improve any opportunity that might present itself for relieving him. The case passed on for several days without material change, save the gradual enlarging of the tumor. He was now visited by his brother, Dr. J. Tremble, of Salisbury, and Dr. H. R. Allen, of Charleston, and after examination, Dr. A. pronounced the case one amenable to treatment, and prescribed *Iodide of potassium* internally, and a plaster of the *Proto-iodide of mercury* externally over the tumor. Disposed to give the patient the benefit of every doubt, we placed him on the treatment at once, and continued it till its inefficiency was demonstrated, and we thought to withdraw it. Nevertheless, Dr. Trower of Charleston being in the city, visited the case with us, and being inclined to corroborate the opinion of Dr. Allen as to its non-malignancy, suggested that we substitute the *Bromide of potassium*, and persist in the treatment a little longer. We did so without any perceptible effect for good whatever. On the contrary, the tumor gradually enlarged, becoming more and more dense, and less sensible to pain on pressure, somewhat moderated and occupying now nearly the entire left half of abdominal cavity. It is proper to say that for some time previous to this our minds had been diverted to the *spleen* as the primitive seat of disease, and that all other organs involved, were so merely as a consequence of the adhesive nature of the malady. There would be, from time to time, several days of febrile excitement, consequent upon adhesive inflammation, during which time he would suffer greatly with acute lancinating pain through the tumor, together with great restlessness. From about the middle of June he suffered somewhat from an inability to swallow fluids, and would usually not be able to get more than a gill of water into the stomach at once, and that not without more than ordinary effort. Nothing unusual occurred in the case until about the 10th of July, when he was seized with sickness at the stomach, and ejected quite a quantity of blood, and soon afterwards discharged blood quite freely from the bowels; otherwise the bowels were quite easily controlled. The treatment was wholly palliative, and the diet such as he most desired, preference being given to that which contained the most nutritive and the least refuse matter. Long continued pressure on the iliac veins began now to induce œdema of the leg on the left side, which soon extended to the abdomen, and, in fact, to give evidence of dropsical effusion throughout all the tissues. All hope of recovery was now abandoned by the patient and his friends, until the evening before he died, when, at the suggestion of some of our well-to-do citizens, he was visited by an old quack from Terre Haute, Indiana, calling himself Dr. Delamater, advertising largely, and claiming to have an extensive practice in Illinois. I was notified of his august presence, and, on my arrival, found that he had already examined the patient and persuaded the friends that there was yet hope, and by knowing winks, nods, and inuendoes, had wrested from beneath the countenance of the now dying yet conscious man, a strange approving smile. Under a protest as forcible as I could deliver on the occasion, I was subjected to the humiliation of seeing the man put on treatment by this old ignoramus — a treatment at once pointless, aimless and foolish, if not criminal. *Aloes*, *Rhei*, and *Mur. ammonia* internally, and blister, tapping, scarification, etc., externally. The patient died the next day, August 9th, from perforation and hæmorrhage of the stomach, as shown by the *post mortem*, which I conducted twenty hours after death, assisted by Drs. Morris, J. W. Dora, Treat, and Girard, a brief synopsis of which I subjoin, from notes prepared by Dr. Dora at the time. *Rigor mortis* not well marked. Upon opening the cavity of the abdomen, the stomach, transverse colon, and, in fact, all the viscera of the cavity were more or less displaced toward the right side, the left being filled by the diseased mass. The appearance of the alimentary canal as exposed, at first view, was rather healthy. It was not diseased except when it was contiguous to the diseased tissue. After we examined and laid that aside, the spleen was found to be almost entirely transformed into a schirrus mass, with scarcely the resemblance of an organ of any kind. Only the inferior portion could be recognized as normal, and that was of a deep purple, and quite dense. The superior portion and body of the organ were entirely metamorphosed, the internal structure being broken down and puriform, and near the superior extremity protruded the diseased substance proper. It emanated from the organ in the form of an excrescence overlapping it and adhering to it, or, rather, involving the substance and becoming a part of the organ itself. The adhesions were very extensive, involving the liver, stomach, transverse and descending colon, duodenum, pancreas, left kidney, diaphragm, anterior walls of the chest, cartilage of the sternum and ribs, intercostal muscles and pericardium. The left lobe of the liver presented evidence of a previous inflammation, was now softened and slightly gangrenous, and adhered by its under surface to the stomach, which was perforated and filled with dark grumous blood. This took place immediately preceding, and was doubtless the cause of death. The coats of the stomach, duodenum, and colon, except at the points of adhesion, were in a fair state of preservation. The pancreas was very much enlarged and carcinomatous. The kidneys were both softened and friable, the right more so than the left. The omentum was entirely metamorphosed, and the transverse meso-colon gangrenous. The mesentery was in a state of congestion, and the mesenteric glands atrophied and gangrenous. The lungs were atrophied, melanotic, friable, and easily torn. The heart and all other organs examined were normal, except, perhaps, that feeble condition of tissue consequent upon defective nutrition. I removed the spleen, and so much of the adjacent tissue as was most completely metamorphosed, and examined it apart from the cadaver. Its physical appearance was somewhat nodulated, of a dull, white color externally, cheese-like in consistence, melanotic internally, and weighed ten pounds. When cut through, a sand-like reaction was apparent, and the divided surface resembled in some degree that of fibro-cartilage. I regret that for want of an instrument of sufficient power, I am unable to give the result of a microscopic examination. In conclusion, it will be remembered that carcinoma of the spleen is a very rare occurrence, and only that our minds had recently been drawn out on the subject of carcinoma, we should have failed, perhaps, to make a diagnosis so easily, but though it be a coincidence rarely met with in a country practice in so short a time, this is the second case we have attended the present year. The other was that of Mr. Philip A. Crow, who died of cancer of the stomach in January last, and upon whose body I conducted a *post mortem* in the presence of Drs. Morris, Dora, and Wilcox, an account of which will be found reported by Dr. J. W. Dora in the JOURNAL for March 1st, 1868. A very striking similarity is found to exist in the more prominent symptoms of the two cases.

